# Changes in Industry Marketing and Research Payments to US Physicians and Teaching Hospitals During the COVID-19 Pandemic

**DOI:** 10.1001/jamahealthforum.2022.3342

**Published:** 2022-09-30

**Authors:** Nishant Uppal, Timothy S. Anderson

**Affiliations:** 1 Harvard Medical School, Boston, Massachusetts; 2 Department of Medicine, Brigham and Women’s Hospital, Boston, Massachusetts; 3 Division of General Medicine, Beth Israel Deaconess Medical Center, Boston, Massachusetts; 4 Center for Healthcare Delivery Science, Beth Israel Deaconess Medical Center, Boston, Massachusetts

## Abstract

This cross-sectional study explores ways that the COVID-19 pandemic was associated with changes in research and marketing payments from pharmaceutical and medical device companies before and during the pandemic.

## Introduction

The COVID-19 pandemic has had wide-ranging effects on health care, including shifting research priorities and reducing physical interactions in clinical and research settings. Research and marketing payments from pharmaceutical and medical device companies (hereafter, industry) are major sources of funding for physicians and teaching hospitals,^1^ but how the COVID-19 pandemic has affected these payments is unknown. In this serial cross-sectional study, we hypothesized that the COVID-19 pandemic would be associated with changes in industry payments in multiple ways, including restrictions on in-person events changing patterns of marketing payments, disruptions in clinical trials leading to lower research expenditures, and, given the increased use of Emergency Use Authorizations, shifts in research expenditures toward products not receiving US Food and Drug Administration approval.^2^

## Methods

We examined trends in industry research and marketing payments from 2018 to 2021 using the Centers for Medicare & Medicaid Services Open Payments database.^3^ Inclusion/exclusion criteria, payment classification, and product type definitions are available in the eMethods in the Supplement.

We calculated inflation-adjusted mean monthly values of marketing and research payments, overall and stratified by recipient, payment category, and product type. We compared mean monthly payment values from January 2018 through February 2020 (prepandemic period) to March 2020 through December 2021 (pandemic period). We conducted interrupted time-series analyses comparing monthly payments in the prepandemic and pandemic periods using ordinary least squares regressions with Newey-West standard errors to account for autocorrelation (eMethods in the Supplement).^4^

Because this study used only publicly available data, it was exempt from institutional review board approval, and informed consent was waived in accordance with the Common Rule (45 CFR §46). Stata, version 16 (StataCorp), was used for all analyses.

## Results

During the 4-year study period, 705 490 physicians and 4631 hospitals received $25.2 billion in research payments and $6.0 billion in marketing payments. Compared with the prepandemic period, mean monthly research payments increased by 5.7% in the pandemic period ($485.8 million to $513.4 million), while marketing payments declined by 38.6% ($161.9 million to $99.5 million) (Figure, A). Growth in research payments was driven by increased payments for biologics and “not specified” product types (Table). Declines were observed among all marketing payment categories (Figure, B and Table).

**Figure.**
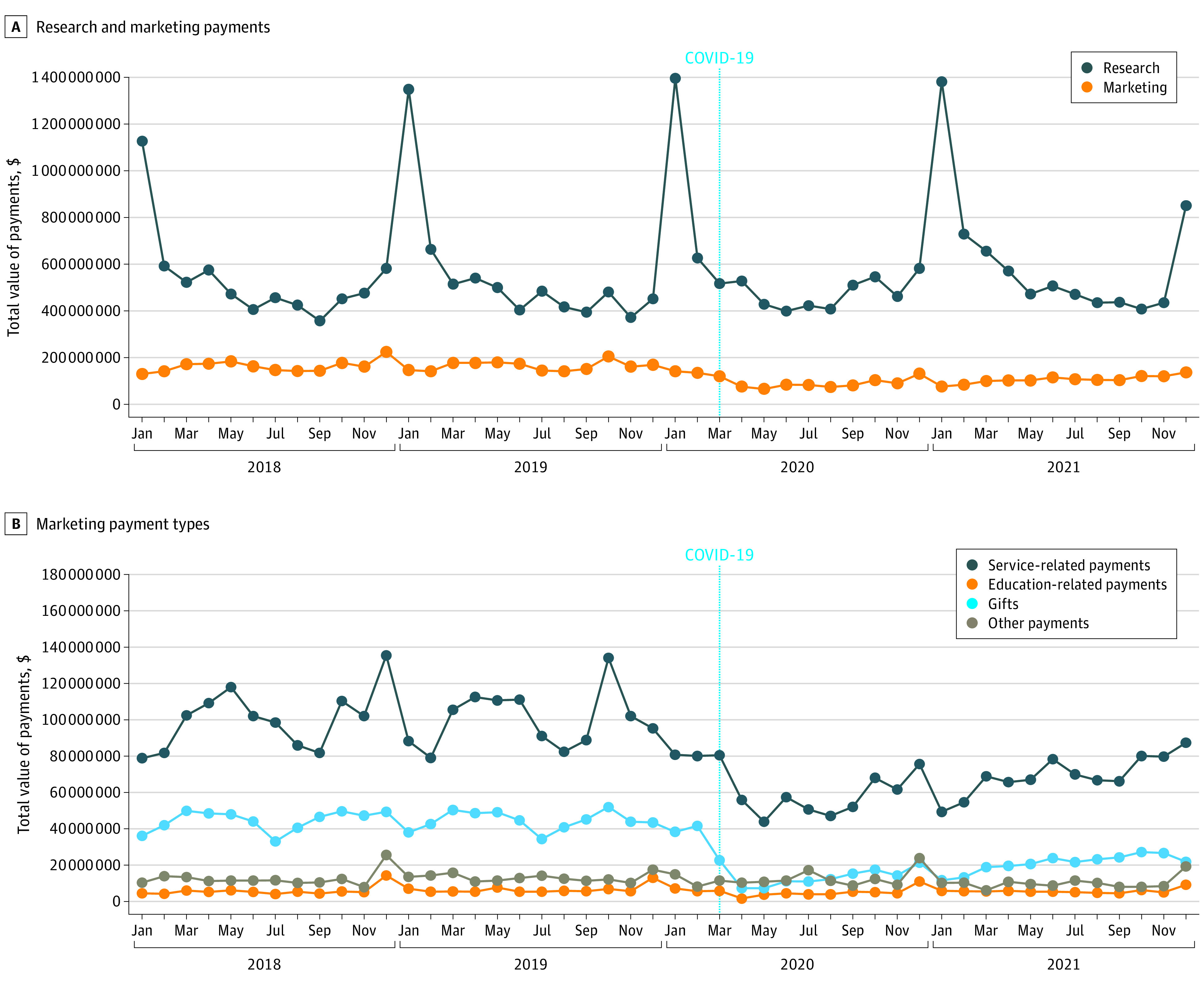
Industry Payments to US Physicians and Teaching Hospitals, 2018-2021 All estimates are reported in 2021 inflation-adjusted dollars using Consumer Price Index data from the US Bureau of Labor Statistics (eMethods in the Supplement). Gifts included payments for food and beverage, entertainment, gifts, and travel/lodging. Service-related payments included payments for consulting, faculty/speaker compensation for accredited or certified continuing education programs, faculty/speaker compensation for nonaccredited or noncertified continuing education programs, educational activities, and honoraria. Education-related payments included payments for classes, activities, programs, or events that involve the imparting of specific knowledge or skills. Other payments included charitable contributions, nonresearch grants, and facility fees for space rental at teaching hospitals.

**Table.** Mean Monthly Changes and Interrupted Time-Series Analysis of Industry Marketing and Research Payments, 2018-2021^a^

Outcome	Mean monthly payments value, millions $			Interrupted time-series analysis, millions $ (95% CI)			
	Prepandemic	Pandemic	Difference, %	Prepandemic slope	Immediate level change at start of pandemic	Pandemic slope	Difference in slopes
Marketing payments^b^							
Overall	161.9	99.5	–38.6	0.0 (–0.5 to 0.6)	–84.6 (–99.2 to –69.9)	1.9 (1.3 to 2.5)	1.9 (1.2 to 2.5)
Physicians	142.0	84.5	–40.5	–0.1 (–0.6 to 0.4)	–78.8 (–92.4 to –65.2)	2.0 (1.4 to 2.6)	2.1 (1.5 to 2.7)
Teaching hospitals	19.9	14.9	–25.1	0.1 (0.0 to 0.3)	–5.8 (–10.0 to –1.6)	–0.1 (–0.4 to 0.2)	–0.2 (–0.6 to 0.1)
Payment category							
Gifts	44.2	17.9	–59.5	0.0 (–0.1 to 0.1)	–33.9 (–36.8 to –30.9)	0.7 (0.6 to 0.9)	0.7 (0.6 to 0.9)
Service related	98.8	64.9	–34.3	–0.1 (–0.5 to 0.4)	–46.3 (–57.2 to –35.3)	1.1 (0.7 to 1.6)	1.2 (0.7 to 1.7)
Education related	6.2	5.4	–13.9	0.1 (0.1 to 0.1)	–3.3 (–4.5 to –2.2)	0.1 (0.0 to 0.2)	0.0 (–0.1 to 0.1)
Other	12.7	11.3	–11.0	0.0 (0.0 to 0.1)	–1.1 (–3.0 to 0.8)	–0.1 (–0.2 to 0.1)	–0.1 (–0.3 to 0.1)
Product type							
Biologic	17.1	9.9	–42.2	0.0 (0.0 to 0.1)	–11.3 (–13.6 to –9.1)	0.3 (0.2 to 0.4)	0.3 (0.1 to 0.4)
Device	48.8	29.2	–40.1	0.1 (–0.2 to 0.3)	–26.7 (–32.9 to –20.5)	0.6 (0.3 to 0.8)	0.5 (0.2 to 0.8)
Drug	55.9	29.4	–47.4	–0.3 (–0.5 to –0.1)	–31.2 (–35.1 to –27.2)	0.7 (0.5 to 1.0)	1.0 (0.7 to 1.3)
Medical supply	0.2	0.3	45.8	0.0 (0.0 to 0.0)	–0.5 (–0.9 to 0.0)	0.0 (0.0 to 0.1)	0.0 (0.0 to 0.1)
Not specified^c^	40.0	30.7	–23.3	0.2 (0.0 to 0.5)	–14.9 (–20.2 to –9.6)	0.3 (0.0 to 0.5)	0.0 (–0.3 to 0.4)
Research payments^d^							
Overall	485.8	513.4	5.7	1.2 (–1.7 to 4.1)	–24.1 (–97.4 to 49.2)	3.8 (–1.7 to 9.3)	2.6 (–3.3 to 8.6)
Physicians	365.5	386.5	5.7	0.8 (–1.4 to 3.1)	–24.2 (–86.8 to 38.4)	4.0 (–0.7 to 8.6)	3.1 (–1.8 to 8.1)
Teaching hospitals	120.4	126.9	5.4	0.4 (–0.4 to 1.2)	0.1 (–18.0 to 18.2)	–0.2 (–1.8 to 1.4)	–0.5 (–2.3 to 1.3)
Product type							
Biologic	93.7	115.0	22.7	–0.6 (–1.3 to 0.1)	–15.7 (–46.7 to 15.3)	4.1 (0.3 to 7.8)	4.7 (0.8 to 8.5)
Device	38.5	30.5	–20.8	0.1 (0.0 to 0.2)	–9.3 (–12.3 to –6.3)	0.0 (–0.2 to 0.2)	–0.2 (–0.4 to 0.1)
Drug	187.1	164.0	–12.3	–1.2 (–2.7 to 0.4)	–9.6 (–41.0 to 21.8)	–0.5 (–1.6 to 0.6)	0.6 (–1.1 to 2.4)
Medical supply	0.1	0.1	–26.6	0.0 (0.0 to 0.0)	0.0 (–0.1 to 0.0)	0.0 (0.0 to 0.0)	0.0 (0.0 to 0.0)
Not specified^c^	166.5	203.9	22.4	2.8 (1.2 to 4.4)	10.6 (–52.4 to 73.5)	0.3 (–4.5 to 5.1)	–2.5 (–7.4 to 2.3)

^a^ All estimates are reported in 2021 inflation-adjusted dollars using Consumer Price Index data from the US Bureau of Labor Statistics (eMethods in the Supplement). The prepandemic period was defined as January 2018 to February 2020, and the pandemic period was defined as March 2020 to December 2021.

^b^ Marketing payments were categorized as gifts, service-related payments, and education-related payments. Gifts included payments for food and beverage, entertainment, gifts, and travel/lodging. Service-related payments included payments for consulting, faculty/speaker compensation for accredited or certified continuing education programs, faculty/speaker compensation for nonaccredited or noncertified continuing education programs, educational activities, and honoraria. Education-related payments included payments for classes, activities, programs, or events that involve the imparting of specific knowledge or skills.

^c^ “Not specified” product type refers to payments made for products that have not yet received US Food and Drug Administration approval, licensure, or clearance for a covered biological, drug, device, or medical supply (according to Open Payments final rule, 42 CFR §402, §403).

^d^ Research payments include funding for clinical trial development and implementation, compensation to providers for research activities, and covered expenses for study participants. Owing to special reporting exemptions for research payments, January payments were excluded from mean monthly payment comparisons, and an indicator variable for January was included in interrupted time-series analyses (eMethods in the Supplement).

Interrupted time-series analyses showed that monthly marketing payments were stable prior to the pandemic, immediately declined at the onset of the pandemic by $84.6 million (95% CI, –$99.2 million to –$69.9 million), and subsequently increased at a monthly rate of $1.9 million (95% CI, $1.3 million to $2.5 million) (Table). Research payments were stable in the prepandemic period and experienced no immediate change at the onset of the pandemic. In the pandemic period, research payments for biologics increased by $4.1 million per month (95% CI, $0.3 million to $7.8 million), while there was not a statistically significant change in payments for other product types.

## Discussion

The COVID-19 pandemic was associated with shifts in the focus of industry payments for research and an immediate and sustained decline in payments for marketing. Research payments were stable during the pandemic despite widespread clinical trial suspensions, which may reflect clinical trial adaptations, including virtual study visits and remote outcome measurement.^5^ Growth in biologics and “not specified” product types likely reflect shifts in research investments toward COVID-19 therapeutics and vaccines.

Marketing payments likely declined as a result of pandemic-related social distancing and visitor restriction policies but rebounded during the pandemic despite simultaneous increases in new COVID-19 cases. Growth in marketing payments during the pandemic may have reflected changes in industry marketing strategies, such as shifting to online medical education and e-detailing.^6^

Study limitations include a reliance on industry reporting, which is not verified by the Centers for Medicare & Medicaid Services, and a lack of reporting on drug samples, payments by companies without marketed products, and payments to nonclinician researchers, medical trainees, and nonteaching hospitals. Further study is needed to understand the downstream effect of large shifts in industry research and marketing payments on innovation and clinical practice.

## Notes

Supplement.
eMethods

eReferences
