# Supplementary material for: Changes in Industry Marketing and Research Payments to US Physicians and Teaching Hospitals During the COVID-19 Pandemic
Source: JAMA Health Forum. 2022 Sep 30;3(9):e223342. doi: 10.1001/jamahealthforum.2022.3342 (PMC9526091; doi:10.1001/jamahealthforum.2022.3342)
Supplement: Supplement. — eMethods eReferences [file jamahealthforum-e223342-s001.pdf]

Supplemental Online Content

Uppal N, Anderson TS. Changes in industry marketing and research payments to US physicians and teaching hospitals during the COVID-19 pandemic. *JAMA Health Forum*. 2022;3(9):e223342. doi:10.1001/jamahealthforum.2022.3342

eMethods
eReferences

This supplemental material has been provided by the authors to give readers additional information about their work.

© 2022 Uppal N et al. *JAMA Health Forum.*

eMethods

Overview of the Open Payments Program

Under the Patient Protection and Affordable Care Act, the Centers for Medicare & Medicaid Services (CMS) developed Open Payments, a national disclosure program for financial relationships in the health care industry.[1] Each year, applicable manufacturers and group purchasing organizations that are engaged in commercial activities related to drugs, devices, biologics, or medical supplies are required to disclose certain payments and transfers of value made to covered recipients, which include physicians, teaching hospitals, and certain non-physician health care providers.[1]

Study Population

Our analysis included industry payments to physicians and teaching hospitals between 2018 and 2021. For each payment, manufacturers are mandated to report the nature and value of the payment, the recipient, and the associated product type. Manufacturers are mandated to report the product type when disclosing payments made to covered recipients, including for products without marketed names or that are not yet covered. Payments related to covered or non-covered products that cannot be categorized as drugs, devices, biologics, or medical supplies are categorized under the "Not Specified" product type.[1]

Payments are categorized by CMS as research payments, general (non-research payments), or ownership and investment payments.[1] We excluded ownership and investment interests and royalty payments, as these payments are typically established by contracts with long-term periods and because Open Payments does not make available information on company policies regarding the distribution of equity, financial leverage, and other fiduciary factors[1] that would be necessary to understand how the value of ownership interests change over time and during the COVID-19 pandemic.

General payments refer to non-research, non-ownership, non-investment related payments. We define marketing payments as general payments and further categorized them into gifts, service-related payments, education-related payments, and other payments. Gifts included payments for

© 2022 Uppal N et al. *JAMA Health Forum.*

food and beverage, entertainment, gifts, and travel/lodging. Service-related payments included payments for consulting, honoraria, faculty/speaker compensation for accredited or certified continuing education programs, and faculty/speaker compensation for non-accredited or non-certified continuing education programs. Education-related payments included payments for classes, activities, programs, or events that involve the imparting of specific knowledge or skills. Other payments included charitable contributions, non-research grants, and facility fees for space rental at teaching hospitals. We excluded 3 additional general payment categories which were added in 2021 and not previously required to be reported (debt forgiveness, long-term medical supply or device loan, and acquisitions). A single outlier service-related payment of $49 million in October 2019 (20.6% of total monthly payment value) was detected and excluded from analysis.

Research payments include funding for clinical trial development and implementation, compensation for providers for research activities, and covered expenses for study participants. CMS additionally requires research payment reporting for non-covered recipient institutions which receive payments on behalf of a covered physician or teaching hospital (for example a university which receives a research grant for which a covered physician serves as the principal investigator) and for non-covered recipient entities such as employees of covered physicians.[2] We categorized indirect payments as payments to physicians based on listed physician principal investigator.

Delayed Research Payment Reporting

Research payments are subject to special reporting rules under Part 403 of Subchapter A of Chapter IV of Title 42 of the Code of Federal Regulations (CFR)[3]; certain research payments pertaining to novel drugs, devices, biologics, or medical supplies may be delayed from publication in Open Payments if they are made under product research and development agreements and/or clinical investigations. When this exemption is granted, reporting can be delayed until the first reporting date of the following year, which typically falls in January. As this reporting exemption applies only to research payments, this likely explains why payment spikes are seen in January of each year for research payments, but not for marketing payments shown in the Figure. Therefore, reported research payments in January are likely to overestimate

© 2022 Uppal N et al. *JAMA Health Forum.*

the value of payments actually made in this month. To account for this, January payments were excluded from mean monthly payment comparisons and an indicator variable for January was included in interrupted time series analyses, as detailed below.

Statistical Analysis

Payments are recorded for each transaction between a manufacturer and a covered entity, which is logged on a specific date. To compare pre-pandemic and pandemic trends, we aggregated payments on a monthly basis. Payments were adjusted for inflation using Consumer Price Index data from the Bureau of Labor Statistics,[4] and all estimates are reported in 2021 inflation-adjusted dollars.

We calculated the mean monthly value of payments overall and stratified by recipient (physician or teaching hospital), payment category, and product type. We then compared mean payment values from January 2018 – February 2020 (pre-pandemic) and March 2020 – December 2021 (pandemic). For research payments, all January payments were excluded from both time periods, given the previously discussed reporting exemption.

Interrupted Time-Series Analysis Methodology

We conducted interrupted time series (ITS) analyses of payments, comparing pre-pandemic (January 2018 – February 2020) and pandemic (March 2020 – December 2021) periods using ordinary least squares regressions with Newey-West standard errors to account for autocorrelation. Analyses were conducted separately for overall marketing and research payments, and by subgroups defined by recipient, payment category, and product type. For research payment analyses, an indicator variable was included for the month of January because research payments peak cyclically in January due to reporting exemptions.

Interrupted time series models were run using Stata 14.1 using the *itsa* command to generate ordinary least squares regression estimates using Newey-West standard errors to address autocorrelation. We initially tested for autocorrelation in models using 0 lags using the Cumby-Huizinga test (Stata package *actest*) and found autocorrelation to be present up to 12 lags

© 2022 Uppal N et al. *JAMA Health Forum.*

(consistent with monthly seasonality in the data). Thus, all models were run with 15 lags to be considered in the autocorrelation structure.[5]

For each outcome, we report the immediate level change at the onset of the COVID-19 pandemic (March 2020), the pre-pandemic and pandemic slopes, and the difference in slopes.[6] Statistical significance was assessed using 95% confidence intervals. March 2020 was considered the observation dividing the preintervention and postintervention time periods, since the United States declared a national emergency due to the COVID-19 outbreak during this month.

© 2022 Uppal N et al. *JAMA Health Forum.*

eReferences

1. Centers for Medicare and Medicaid Services. Open Payments. https://openpaymentsdata.cms.gov/. Updated June 30, 2021. Accessed July 9, 2022.
2. Centers for Medicare and Medicaid Services. Open Payments: Frequently Asked Questions (FAQs). https://www.cms.gov/OpenPayments/Downloads/open-payments-general-faq.pdf. Updated April 5, 2022. Accessed July 20, 2022.
3. eCFR :: 42 CFR 403.910 -- Delayed publication for payments made under product research or development agreements and clinical investigations. Accessed July 10, 2022. https://www.ecfr.gov/current/title-42/chapter-IV/subchapter-A/part-403/subpart-I/section-403.910
4. Consumer Price Index (CPI) Databases. U.S. Bureau of Labor Statistics. https://www.bls.gov/cpi/data.htm. Accessed August 3, 2022.
5. Linden A. Conducting Interrupted Time-series Analysis for Single- and Multiple-group Comparisons. *Stata J*. 2015;15(2):480-500.
6. Kontopantelis E, Doran T, Springate DA, Buchan I, Reeves D. Regression based quasi-experimental approach when randomisation is not an option: interrupted time series analysis. *BMJ*. 2015;350:h2750.

© 2022 Uppal N et al. *JAMA Health Forum.*
